# A Connectivity-Based Eco-Regionalization Method of the Mediterranean Sea

**DOI:** 10.1371/journal.pone.0111978

**Published:** 2014-11-06

**Authors:** Léo Berline, Anna-Maria Rammou, Andrea Doglioli, Anne Molcard, Anne Petrenko

**Affiliations:** 1 Université du Sud Toulon-Var, Aix-Marseille Université, CNRS/INSU/IRD, Mediterranean Institute of Oceanography (MIO), La Garde, France; 2 Aix-Marseille Université, CNRS/INSU/IRD, Mediterranean Institute of Oceanography (MIO), Marseille, France; 3 CNRS, Laboratoire d'Océanographie de Villefranche, Villefranche-sur-Mer, France; 4 Université Pierre et Marie Curie, Paris 6, Laboratoire d'Océanographie de Villefranche, Villefranche-sur-Mer, France; Technical University of Denmark, Denmark

## Abstract

Ecoregionalization of the ocean is a necessary step for spatial management of marine resources. Previous ecoregionalization efforts were based either on the distribution of species or on the distribution of physical and biogeochemical properties. These approaches ignore the dispersal of species by oceanic circulation that can connect regions and isolates others. This dispersal effect can be quantified through connectivity that is the probability, or time of transport between distinct regions. Here a new regionalization method based on a connectivity approach is described and applied to the Mediterranean Sea. This method is based on an ensemble of Lagrangian particle numerical simulations using ocean model outputs at 1/12° resolution. The domain is divided into square subregions of 50 km size. Then particle trajectories are used to quantify the oceanographic distance between each subregions, here defined as the mean connection time. Finally the oceanographic distance matrix is used as a basis for a hierarchical clustering. 22 regions are retained and discussed together with a quantification of the stability of boundaries between regions. Identified regions are generally consistent with the general circulation with boundaries located along current jets or surrounding gyres patterns. Regions are discussed in the light of existing ecoregionalizations and available knowledge on plankton distributions. This objective method complements static regionalization approaches based on the environmental niche concept and can be applied to any oceanic region at any scale.

## Introduction

The ecoregionalization of the ocean is useful for scientific research, conservation and management of the marine environment and marine resources. For instance, ecoregionalization is needed to extrapolate punctual or transect data to broader areas and to target specific regions for interdisciplinary research (as in the Mediterranean Sea, [1]). Conservation and management goals range from selecting areas to protect [2] to defining fisheries zones or zones for monitoring and mitigating marine pollution.

To date, several approaches of ecoregionalization were used depending on the data at hand [3]. The taxonomic approach is based on species distributions and identifies areas of broadly similar assemblage of species [4]–[6]. The ecological approach is based on habitat characteristics; it separates areas of similar seasonal cycles of physical and biogeochemical variables [7]–[10]. This approach benefited from the nearly continuous coverage of satellite data. Lastly, the integrative approach is a combination of both taxonomic and ecological approaches that takes into account both the habitat and the species inhabiting it [11].

However, in the marine environment the species distribution not only results from selection by the local environment but also from dispersal of propagules and adults organisms (e.g. the metapopulation concept of Levins [12], [13]). Therefore an ecoregionalization based on dispersal by ocean circulation is needed; recent studies start taking into account dispersal in defining management units [14]. However it was never achieved quantitatively at basin scale. Today this is possible, as widely available ocean circulation models provide 3 dimensional, time varying, realistic and consistent depictions of oceanic currents at basin scale. The goal of this paper is to present a regionalization method based on connectivity, assessed from ensemble Lagrangian simulations using ocean circulation model velocity outputs.

This method is applied to the Mediterranean basin, which is a target region for spatial planning owing to its high level of endemism and high biodiversity [15]. Surface circulation shows a complex pattern of larger and smaller gyres, driven by the entrance of Atlantic water at Gibraltar Strait [1], local meteorology and bathymetry. The oligotrophy increases toward the East, but productive spots also exist over shelves and deep mixing areas, thus creating a significant heterogeneity in ecosystem functioning and habitats.

## Materials and Methods

**Figure 1:**
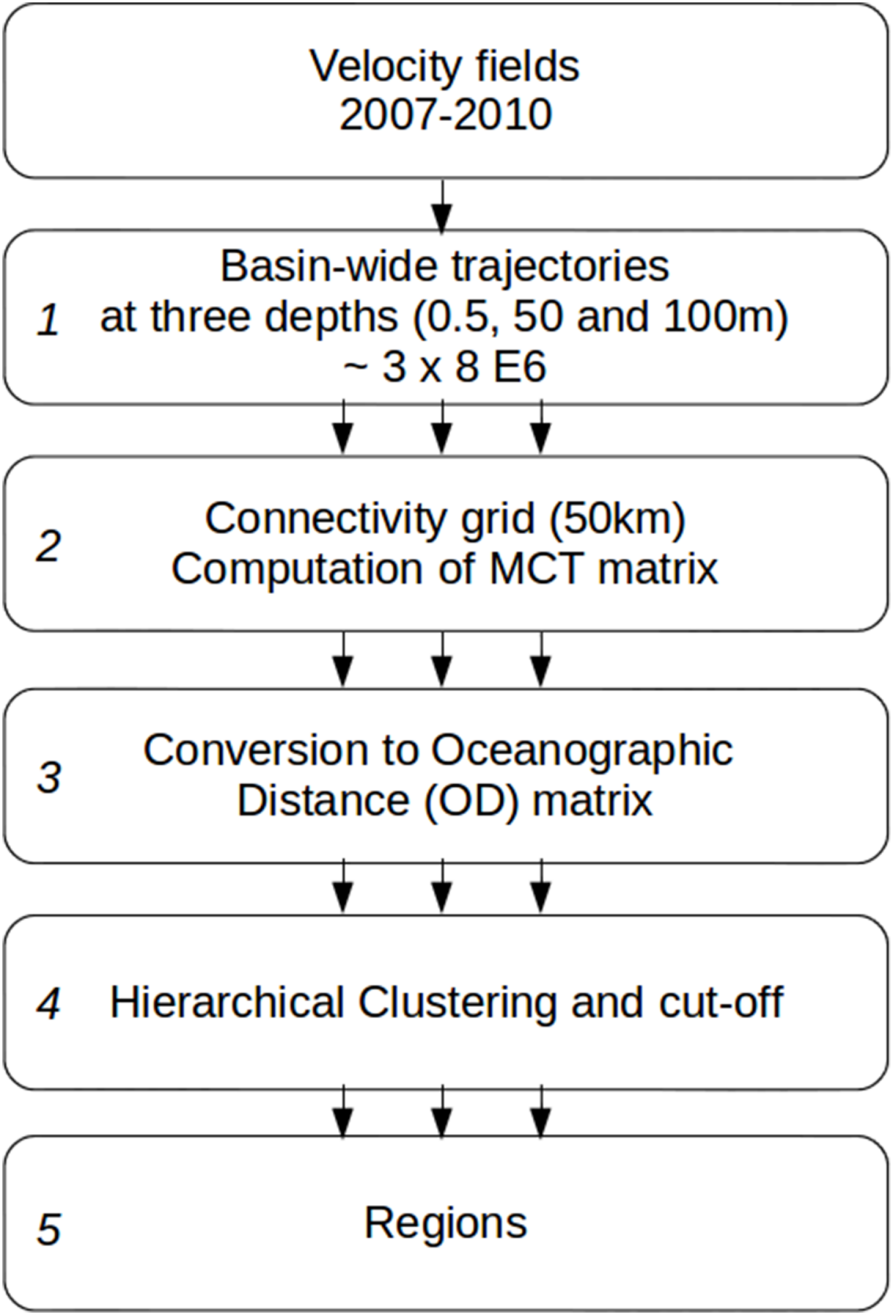
Schematic of the steps of the regionalization method. Note that steps 2 to 5 are repeated using trajectories at the 3 depths separately, shown with the three arrows, and then using them altogether.

The general outline of the method is as follow (Fig. 1): Lagrangian trajectories are computed from ocean circulation model velocity outputs for particles seeded over the whole model domain at three depths (0.5 m, 50 m and 100 m). The domain is divided into a regular grid (hereinafter connectivity grid) and the trajectories are used to derive the mean connection time between every pair of grid cell. In this way a mean connection time matrix is obtained and then transformed into an oceanographic distance matrix, used as input to a hierarchical clustering algorithm. Finally clustering produces a partition of the domain.

Daily outputs velocity fields for four years (2007–2010) were taken from the configuration PSY2V3 of the operational system MERCATOR OCEAN [16]. The PSY2V3 configuration covers the North Atlantic ocean and Mediterranean Sea, and is based on the NEMO-OPA primitive equations code [17] with assimilation of observed data (satellite and in situ). Here, only the domain subset covering the Mediterranean Sea was used. Daily surface forcing are provided by ECMWF [18]. The velocity components are distributed in an Arakawa C type grid [19]. The horizontal resolution is 1/12° (∼8 km) and there are 50 fixed vertical levels with higher resolution at the surface. The vertical mixing is described by a TKE closure scheme [20] and the advection by a TVD 2nd order centered scheme [21].

The trajectories followed by numerical particles were calculated offline with the Lagrangian diagnostic tool ARIANE [22]. The trajectories only result from the horizontal advection at three depths (0.5 m hereinafter called surface, 50 m and 100 m) chosen to represent the transport in the epipelagic layer. No vertical velocity was considered to keep particles in the 0–100 m range. The one year integration time was chosen to allow particles to cover the whole basin and therefore quantify basin scale connectivity and to keep computation time reasonable. Particles were seeded every 10 km on a regular square grid covering the whole domain, totaling 25,646 initial positions for surface depth and 23,770 for depths 50 and 100 m because the domain is smaller. Particles were seeded every 3 days from the 1st to the 25th of every month, from January 1^st^ 2007 to December 25^st^ 2009 in order to fully sample the variability of the circulation. This represents a total of 8,309,304 particles for surface depth, respectively 7,701,480 for depths 50 and 100 m. The choice of 10 km and 3 days is a compromise between matching the horizontal resolution of the model, taking into account mesoscale processes and keeping an affordable computing time of resulting trajectories. We thus obtained three ensembles of trajectories, one per depth.

In order to quantify the connections over the model domain, the domain was divided into grid cells of 50 km×50 km on a regular square grid, the connectivity grid, with a total of 1095 cells covering only regions with depths greater than 100 m. The 50 km resolution is sufficient to keep a reasonably realistic coastline while being suitable with the seeding density chosen. Thus each connectivity grid cell contains 5*5 = 25 particles for each initial seeding date, except grid cells including land that contains less particles.

To quantify the connectivity between each grid cell, we used the Mean Connection Time, hereinafter MCT. Defining T(i,j) as the transit time from grid cell *i* to grid cell *j*, MCT(i,j) was computed as
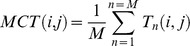



*M* being the number of particle transitioning from *i* to *j*. Note that for each trajectory, all intermediate transitions were used to compute the MCT. The sensitivity of MCT to the number of particles was tested. The suite of MCT matrices converged when the number of particles was greater than 6,000,000, therefore we considered that 8,309,304 particles and respectively 7,701,480 particles for depths 50 and 100 m were sufficient to obtain a robust MCT matrix. Moreover, to keep MCT robust, it was computed only when *M* was greater or equal to 50. Four MCT matrices of size 1095×1095 were computed: one MCT matrix from each ensemble of trajectories (MCT_0_, MCT_50_, MCT_100_ for 0.5, 50 and 100 m depths trajectories respectively) and also one MCT matrix using the three ensembles together (MCT_3depths_).

Not all grid cells of the domain were connected within one year, especially remote cells (e.g. Northern Aegean and Gibraltar Strait). Thus the resulting MCT matrices had gaps (from 37% to 56%). These gaps are a problem for the steps of computing the oceanographic distance and applying hierarchical clustering on it. Therefore a gap filling procedure was introduced as follows (see Appendix S1):

○ For each unconnected pair of grid cells i-j, we looked for grid cells k so that i-k and k-j pairs are connected. There must be at least 50 grid cells k as for *M*.

○ Then we computed MCT(i,j) for pair i-j as the sum of the MCT(i,k) and MCT(k,j), averaged on all existing cells k, and filled the MCT(i,j) value in the matrix.

After 3 iterations of this procedure, each MCT matrix was filled. The resulting MCT values ranged from 10 days to 3000 days. This gap filling procedure avoided the very long integration time (>8 years) needed if we were to fill the whole MCT matrices from original trajectories alone.

This led to four full MCT matrices, which are asymmetric since the time to go from *i* to *j* is not equal to the time to go from *j* to *i*. Then the oceanographic distance (OD) was defined after [23] as the minimum of the two MCT values associated to each pair of grid cells *i* and *j* (travel from *i* to *j* and return travel from *j* to *i*). We chose the minimum value as it corresponds to the fastest route of transport which is also the shortest in length.
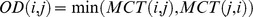



This gave four symmetric matrices, (OD_0_, OD_50_, OD_100,_ OD_3depths_) where all diagonal terms (autoconnection time) were set to zero.

**Figure 2:**
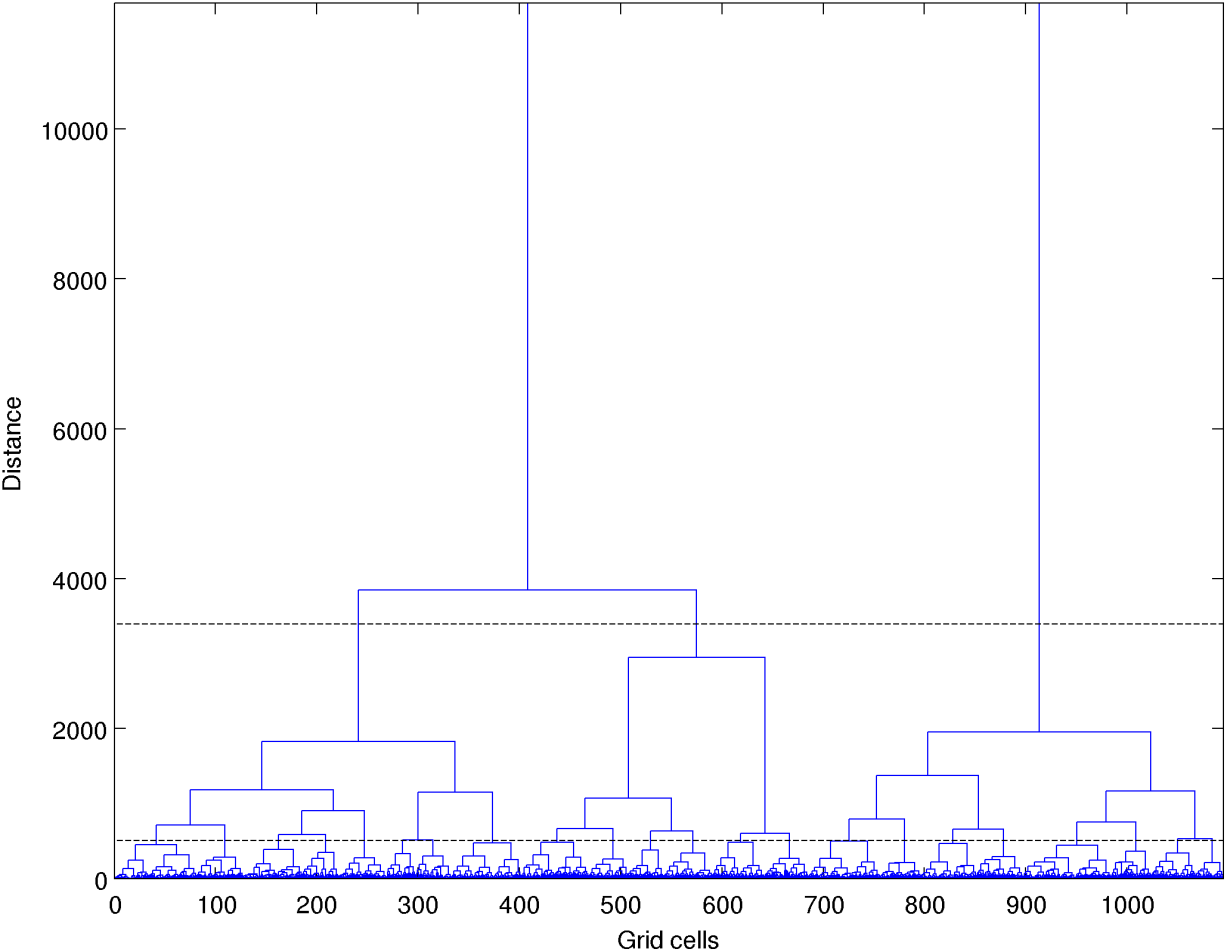
Cluster dendrogram of the oceanographic distance matrix OD_3depths_ using the flexible linkage. Horizontal black lines show the cut-off values for 3 and 22 clusters.

**Figure 3:**
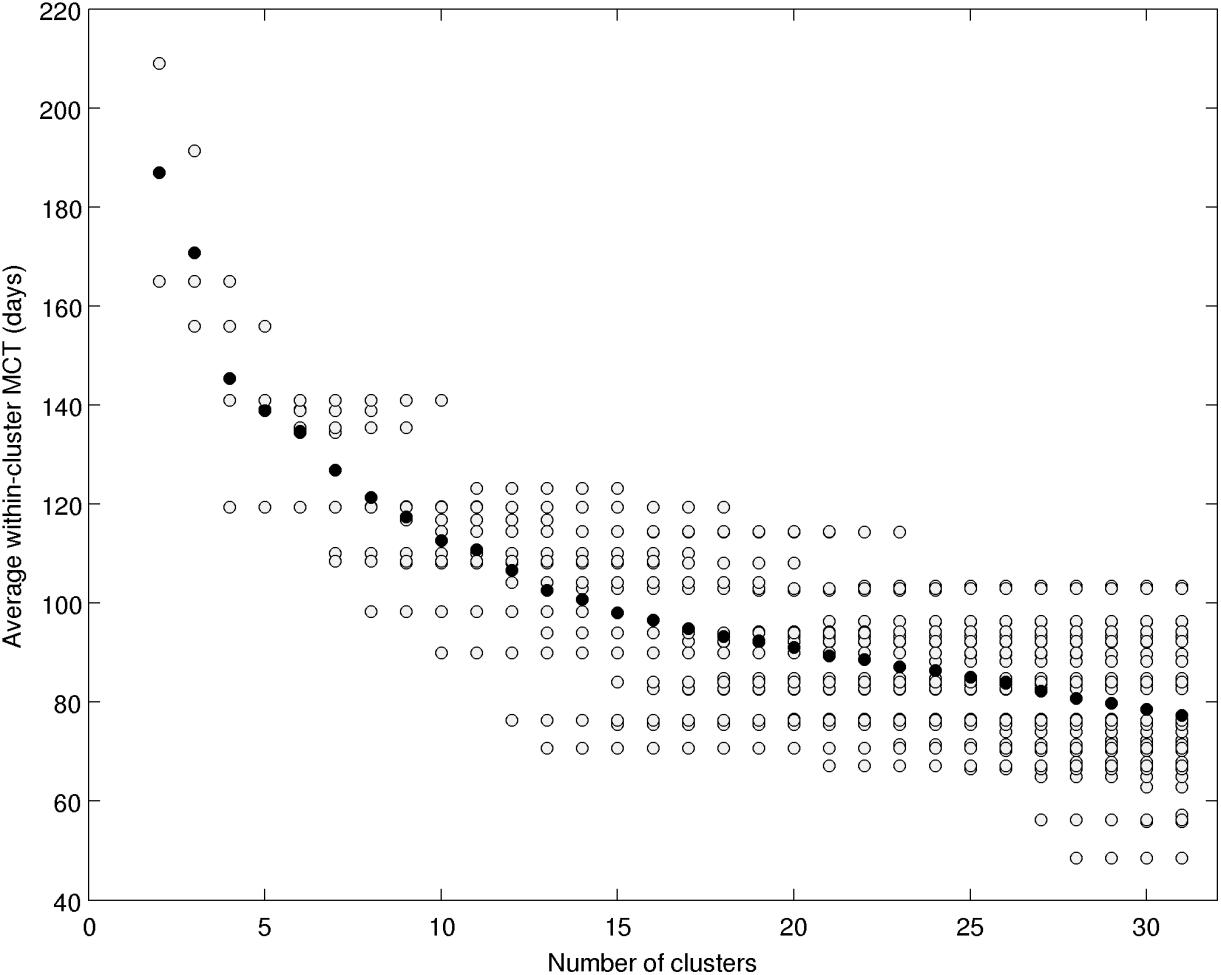
Within cluster mean connection time as a function of the cluster number for MCT_3depths_. White dots are the mean for each cluster, black dots are the mean over all clusters.

Finally hierarchical clustering analysis was applied on each of the oceanographic distance matrix. This method has proved to be robust in the classification of atmospheric wind data (e.g. [24]) and hydrological data (e.g. [25]). Hierarchical clustering assigns grid cells to different clusters in a way that each grid cell belongs to only one cluster [26], and each cluster belongs to a larger cluster (Fig. 2). The grid cells are grouped according to their similarity, which here is the oceanographic distance. Thus there is no distance metric applied as in usual clustering exercises. During each sequence of the clustering algorithm, the distances between the new clusters formed and the other grid cells are computed. This step requires a linkage criterion to be defined. Here we used the flexible [27] and Ward linkages [28]. WPGMA linkage was also tested ([27]) but flexible and Ward best balanced the dendrogram**.** For a given cut-off level of the dendrogram, we obtained a partition of the grid cells in a certain number of clusters, which is, in the spatial domain, a regionalization. Each cluster corresponded to a region on the connectivity grid whose contours were identified. Finally for each cluster, the within-cluster MCT was computed and plotted as a function of the number of clusters from 2 to 31 (Fig. 3).

**Figure 4:**
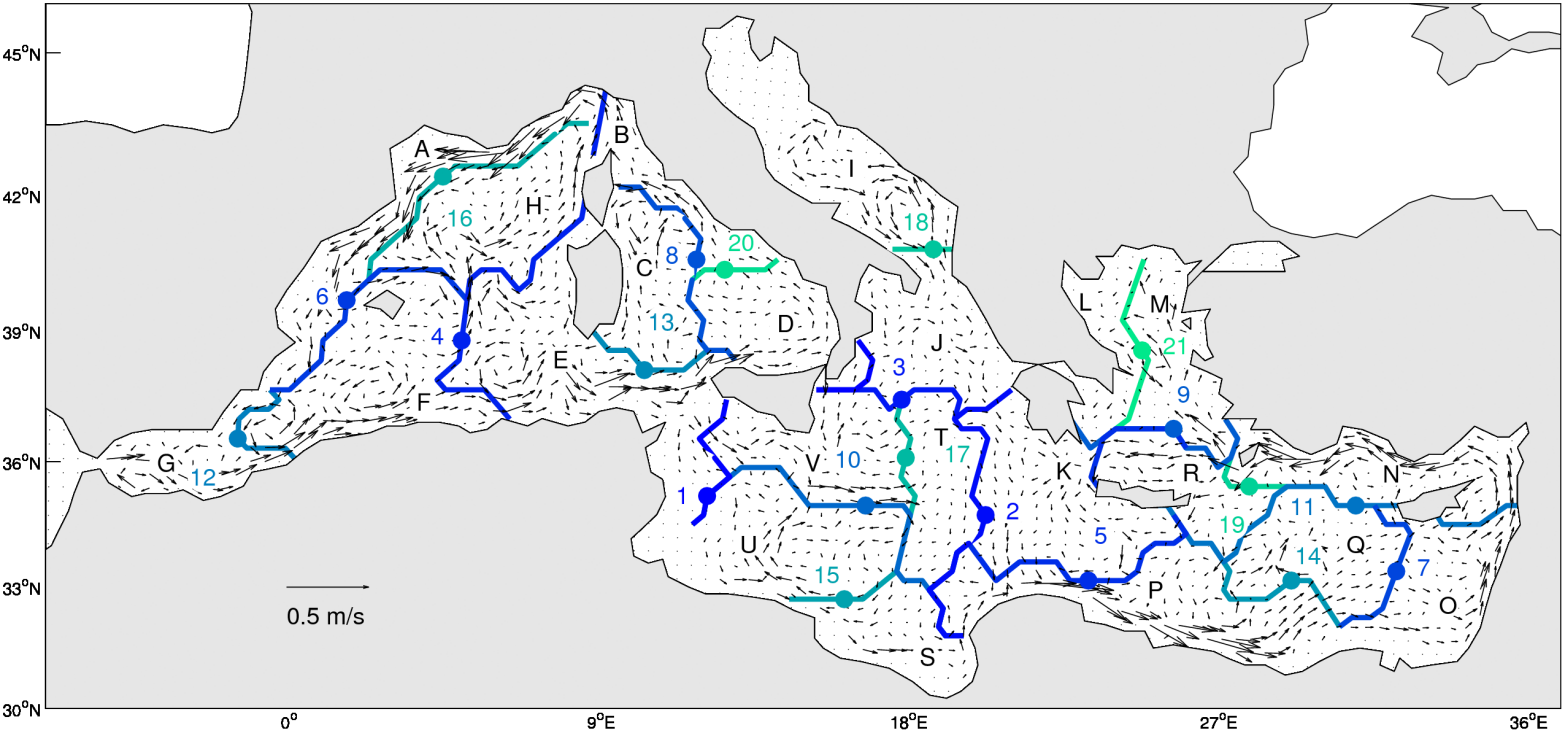
Map of the 21 clusters boundaries obtained from clustering of the oceanographic distance matrix OD_3depths_ using the flexible link. Each boundary is colored and numbered according to the cut-off distance on the dendrogram (from blue – high distance- to green- low distance). Each region is identified by a letter from A to V. The velocity from the circulation model, averaged for the 4-year (2007–2010) and the 3 depths is overlaid as black vectors.

Our “best estimate” regionalization was computed using flexible link and the matrix OD_3depths_, built from the complete ensemble of trajectories (Fig. 4). We also computed one regionalization for each of the three depths and two linkages (6 cases). To assess the sensitivity of the regionalization results to the linkage and depth used, we computed the boundary stability, which is simply the local frequency of occurrence of a boundary in the spatial domain among the 6 cases, as defined in [29].

The choice of the optimal cut-off level and number of cluster is not straightforward here, because the distance matrix (OD) is not computed with a distance metric applied to a given dataset. Thus, usual criteria based on dataset variance within clusters cannot be used (e.g. [30]) because there is no dataset. Instead we took a simple approach comparing results from Ward and flexible linkage. For each partition into n clusters, we compute the proportion of cells classified in the same cluster with Ward and flexible (see Appendix S2). This proportion increases from 82%, to 88% from for n = 2 to n = 6 clusters, then drops to values <70% for n>6. Therefore we consider that the optimal cluster number is 6 as it gives more information while keeping consistent results among the two linkages. However, as no absolute criterion is available, we show the maximum number of clusters that we can interpret, which is 22 clusters. The clusters above 22 require detailed regional information to be interpreted, which is beyond the scope of this study.

## Results

When the number of clusters increases, the within-cluster MCT diminishes, as well as the size of each region (Figs. 3 and 4). The average MCT ranges from 188 days for 2 clusters to ca. 90 days for 22 clusters.

**Figure 5:**
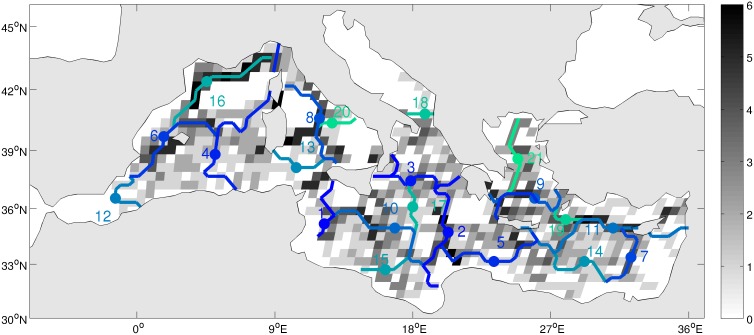
Map of the boundary stability (gray scale) derived from the 6 cases of clustering (3 depths×2 linkages). Boundary stability is defined as the number of occurrence of a boundary in each grid cell among the 6 cases. Boundaries are overlaid as in figure 4.

On the basis of our interpretation of regions with respect to circulation, we retained 22 clusters (Fig. 4). The boundaries of each region were identified and colored according to the number of cluster obtained varying the cutoff distance from 10,000 (2 clusters) to 507 (22 clusters). The boundary #1 partly cuts the Sicily Strait (Fig. 4) and separates the Western and Eastern basins. The boundary #2 isolates Levantine basin from Ionian Sea and Adriatic Sea. The boundary #3 isolates the northern Ionian and Adriatic Sea from Southern Ionian. Then boundary #4 separates the Western basin into a western and an eastern part. The boundary #5 isolates the Levantine basin plus a part of AW current off Lybia from the Aegean Sea. The boundary stability map (Fig. 5) shows that some of the boundaries shown on Fig. 4 are stable (e.g. boundary #7, 11, 16) while others are variable in position or occurrence (e.g. boundary #4). Also, some boundaries (e.g. #2, 6, 8) have only a portion that is stable.

Then considering the 22 regions, the Western basin is separated into eight regions; regions A and B in the Northern part of the basin, G, F and E in the South and C, D that contains the Tyrrhenian sub-basin, region H at the center. In the Eastern basin, the Adriatic Sea is one region I. The Ionian Sea is separated into regions J, V, T at the center and K to the east, with U and S along the coasts of Libya and Tunisia. The Aegean Sea is divided into two regions, M in the East, L in the West. The Levantine basin has four regions: two coastal regions N and O, one southern region P and one center region Q. Considering only stable boundaries, the Western basin only has 5 regions. The Eastern basin has few continuous boundaries, only 4 regions are delimited (Adriatic, South of Sicily Strait and regions U and O).

## Discussion

The boundary stability shows that the majority but not all boundaries are robust to changes in linkages and depths. Often, linkages or depth changes can produce minor shifts in boundary position, hence reducing boundary stability as defined here. When a boundary is not stable, it means that either the circulation is variable, either it is located in a region where the distance (OD) among grid cells is small thus the boundary position varies according to the overall content of each cluster. Thus the boundary map must be analyzed jointly with the boundary stability to assess our regionalization.

### 1. Regions reveal circulation patterns

First the meaning of the regions obtained needs to be explained. One region contains grid cells that are connected at shorter time scale with each other than they are to the grid cells of the other regions. In the following, the relationship between the clusters boundaries, their stability and the circulation is examined in detail in comparison with the model average velocity fields (Fig. 4) and literature.

The hierarchy of cluster boundary is in good agreement with the surface general circulation scheme proposed by Millot et al. [31], their figure 2. Boundary #1 separates the Western and Eastern basins at the Sicily strait, boundary #2 isolates the Eastern Levantine, then boundary #3 the Adriatic Sea together with the northern part of the Ionian. Boundaries are often parallel to the mean velocity field. For instance boundary #16 is parallel to the Northern Current, boundary #11 parallel to the Asia Minor Current. Boundary can also separate two currents branches (the ATC along Tunisia and the AIS along Sicily, for part of boundaries #1 and 10, see [32]). This illustrates the barrier role of semi-permanent jets in the ocean. However, this is not always the case (e.g. boundary #1 at the Sicily Strait, boundary #18 at Oranto Strait). This can occur as the MCT matrix was computed from the time varying flow field, not from the mean field shown here and because each cluster is separated according to its overall distance with other clusters.

In the Western basin, boundary #16 is associated to the path of the Northern Current [31] and is the most stable. The boundary #6 from Spain to the Baleares follows approximately the Balearic front and is also rather stable. The Tyrrhenian Sea contains regions B, C, D with partly stable boundaries. Region C east of the Strait of Bonifacio contains the wind induced cold recirculation identified by [31], which is a potential dense water formation zone [33]. The Southern region G is restricted to the Alboran Sea.

In the Eastern basin, the Ionian Sea has two Southern regions U and S. Boundary #10 follows the Sicilian current of AW and region U contains the area of accumulation of eddies of the Ionian Sea [31]. The region V can correspond to the meandering stream identified by [34] or considered as interannual variability by [31]. The South-eastern Levantine has a region O with a stable boundary #7. Region O corresponds to the eddy accumulation zone ∑LE following [35]. The Asia Minor current along the Southern coasts of Turkey is captured in region N and has a stable boundary #11. Finally, the Aegean Sea is divided into an Eastern region M fed by AW and a North-Western region L fed by Black Sea outflow waters.

Some regions are virgin of any boundaries (Fig. 5), like the center of Gulf of Lion, the Alboran Sea, the Eastern Tyrrhenian Sea, the Northern Adriatic Sea, South of Greece, the South-East of the Levantine basin. This means that these regions are intraconnected at a time scale of less than ca 90 days (see Fig. 3).

Thus this regionalization reveals known circulation patterns and summarizes them in a way that complements the simple average velocity field analysis. It can be used to quantitatively compare the circulation patterns from contrasted periods or from different models.

### 2. Some boundaries coincide with major environmental boundaries and range limits of zooplankton assemblages

The identification of regions close to each other, not geographically but in terms of oceanographic connections, should help understanding the spatial distribution of properties that are passively transported by currents, such as conservative physical properties, or planktonic organisms living in the surface layer (epipelagic).

First, boundaries emerging from circulation alone often match major discontinuities in variables describing the environment. For instance a strong latitudinal salinity gradient exists near the Balearic Islands, close to our boundary #6. However, our boundary #6 coincides with the Balearic Current but not to the Balearic salinity front, located more to the South [36]. Our boundary #16 coincides with a temperature and salinity front in the Ligurian Sea, and also in phytoplankton biomass (Fig. 1 in [10]). Off the Catalan coast, boundary #16 is consistent with the alongshore distribution of fish larvae [37], although located more offshore. Also, boundary #18 south of Adriatic Sea coincides with salinity fronts as seen in MEDATLAS [38]. This results from the dynamic links between density gradients and surface currents. The boundary #21 found in the Aegean Sea parallels the front in phytoplankton biomass [10]. At the Sicily strait, corresponding to our boundary #1, a boundary was also found by [9] (their figure 2) based on a clustering of sea surface temperature and ocean color data.

Within our regions, planktonic organisms are connected at shorter time scales than between regions. Thus hydrodynamical boundaries can become faunistic boundaries as suggested by Gaylord and Gaines [39] for larvae of benthic organisms. Given the spatial resolution, the MCT can correctly resolve connections of plankton organisms with a life cycle greater than 10 days, such as most zooplankton species [40]. Indeed, consistent with boundary #6 north of the Balearic Islands, a boundary exists between Atlantic zooplankton species to the South and Mediterranean species to the North [41], [42]. Also, consistent with our boundaries #1 and #2, differences in zooplankton species composition between Eastern and Western basin were reported by several authors ([43] and references therein, [44]) although the spatial resolution of zooplankton data is generally not sufficient for accurately locating boundaries.

Ecoregions drawn qualitatively from expert knowledge of species assemblages ([45] their figure 2) also distinguish Atlantic-water regions including our region G, a Northern Current region including our region A, three Adriatic regions, one Aegean Sea region including our regions L and M, and two large zonal Eastern basin regions mostly consistent with boundaries #5 and #11.

However, for living organisms such as zooplankton, circulation alone is not sufficient to explain the distribution of a given species as it is adapted to its environment, in particular to a temperature range, e.g. [46]. Thus within our connected regions environmental conditions will restrict a species distribution to its specific preferendum, i.e. its ecological niche**.** Moreover, we deal with particles in the 0–100 m layer, which only properly represent epipelagic zooplankters dispersal.

### 3. How to use this regionalization?

To use this regionalization, the question of the number of clusters to retain will arise. With our approach, no existing criterion is available to define the optimal number. However the number of clusters can be chosen based on the time scale we are interested in, as regions isolated at a given time scale become connected at a larger time scale. Therefore the time scale of interest defines the appropriate cut-off distance and the resulting cluster number and sizes (Fig. 3). For instance, one can look for the scale of dispersal of planktonic larvae and hence consider the Pelagic Larval Duration (PLD) time scale. A PLD of 120 days (e.g. a crustacean as spiny lobster *Palunirus elephas* [47]) gives an adequate cluster number of ca 8. For a PLD of ca. 70 days (e.g. a labridae fish as *Lipophrys trigloides* [48) the adequate cluster number is ca 30. The lower bound time scale we can address with the present regionalization (∼10 days) is set by the spatial resolution of our connectivity grid. Shorter time scales could be achieved with a finer connectivity grid.

Few existing studies can be compared to our regionalization because the approach is original. In the Mediterranean Sea, Andrello et al. [49] obtained clusters of coastal marine protected areas (MPA) based on their connectivity assessed by Lagrangian simulations. Although the velocity fields, Lagrangian simulations set up and clustering method are different, we can compare the overall grouping obtained (their figure 5-A). Considering only clusters containing several MPAs (8 clusters out of 38), their clusters are mostly contained within single regions and do not spread across several regions. Exceptions occur in the Northern Ligurian Sea and Ionian Sea with MPAs located very close or even onto our regions’ boundaries. This probably results from the difference in the input velocity fields and subsequent connectivity quantification.

This new regionalization method quantifies the dispersal range of organisms, This dispersal dimension was shown to explain species distribution (e.g. [50]) and is thus critically needed [51]. This approach complements the usual regionalization methods rooted in the environmental niche concept (e.g. [9], [10]). For instance, the Chl-a based regionalization from [10] reflects the regime of nutrients inputs and stratification, thus they are not directly linked to surface circulation patterns. Adding our connectivity-based regionalization helps understanding the types of environment that plankton is facing, through passive horizontal transport, vertical mixing and production processes. Practically, our OD matrices could be used as a constraint during the clustering of Chl-a, as for chronological clustering [52].

Also, our regions illustrate why plankton organisms may be encountered outside their optimum range (plankton expatriates, e.g. [53]) and where transport-driven fluctuations of plankton communities are expected. Indeed fluctuations of region boundaries may produce large biogeographic fluctuations noticeable at fixed points (e.g. [54], [55]). Regions can also help tracking invasions of exotic organisms, for instance the so-called lessepsian species coming from the Suez Canal [56]. Apart from living organisms, our regions could be used to quantify areas of dispersion of pollutants coming from ships or land sources [57].

Finally this regionalization is useful as a framework to interpret the genetic differentiation of a given species sampled throughout the Mediterranean (e.g. [58]). Further, our approach could be used to define a priori units for grouping existing MPA or set up new MPA (e.g. [59] for the Gulf of Lions), as envisioned in the EU Integrated Project COCONET (www.coconet-fp7.eu).

### 4. Perspectives

The regionalization proposed here will eventually be compared to an ongoing biogeochemistry-based regionalization [60], and to zooplankton species distribution as available in database COPEPODS [61].

Concerning the methods, several points can be made. With a similar approach but shorter simulations, we can explore the seasonal variability of clusters boundaries that may be significant [62]. Here we used hierarchical clustering to extract clusters from the oceanographic distance, but clusters could also be computed with other methods such as graph theory that uses the asymmetry of the connectivity matrix (e.g. [49], [63]). Finally, this method was applied to the Mediterranean Sea but it can be applied anywhere, at any spatial scales as long as accurate and long term model velocity outputs are available.

## Supporting Information

Appendix S1 Method to fill the gap of the MCT matrix.
(DOC)

Appendix S2 Method for choosing the optimal cut-off distance.
(DOC)
