# Supplementary material for: A Connectivity-Based Eco-Regionalization Method of the Mediterranean Sea
Source: PLoS One. 2014 Nov 6;9(11):e111978. doi: 10.1371/journal.pone.0111978 (PMC4222956; doi:10.1371/journal.pone.0111978)
Supplement: Appendix S1 — Method to fill the gap of the MCT matrix. (DOC) [file pone.0111978.s001.doc]

**Appendix S1: Method to fill the gap of the MCT matrix**

|  | 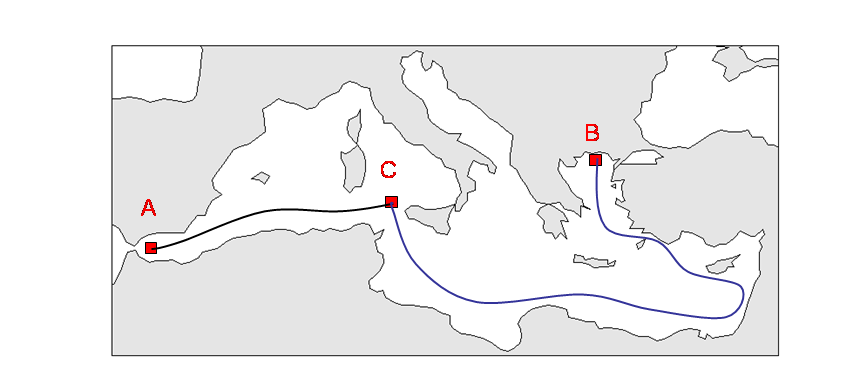 |
| --- | --- |
| A | B |

Figure 1 A: schematic MCT matrix with gaps in white. B: schematic trajectories from Gibraltar Strait to Aegean Sea

The initial MCT matrix has gaps (fig 1A).

1. Here the value MCT_AB_ is not known because there is no one year trajectory that goes from Gibraltar Strait to Aegean Sea (fig 1B).
2. However, intermediate trajectories exist, that go from A to C, and from C to B. So we know MCT_AC_, and MCT_CB_ then we can compute MCT_AB_=MCT_AC_+MCT_CB_.

In the algorithm, we repeat these two steps for each missing value and we fill the missing value only if there are 50 or more intermediate cells.
