# Supplementary material for: A Connectivity-Based Eco-Regionalization Method of the Mediterranean Sea
Source: PLoS One. 2014 Nov 6;9(11):e111978. doi: 10.1371/journal.pone.0111978 (PMC4222956; doi:10.1371/journal.pone.0111978)
Supplement: Appendix S2 — Method for choosing the optimal cut-off distance. (DOC) [file pone.0111978.s002.doc]

**Appendix S2: Method for choosing the optimal cut-off distance**

Figure 2 Dendrogram tree from OD_3depths_ using Flexible (left) and Ward linkage (right)

1. Dendrogram tree are computed from the two linkages Flexible and Ward.
2. For n = 2 to 22,
   1. Cut each dendrogram to retain n clusters
   2. Create two vectors CV_flexible and CV_ward of size m, m being the number of grid cells
   3. For each vector, assign to each element of CV its cluster value according to the dendrogram
   4. Compute the difference D=CV_flexible-CV_ward
   5. Count the proportion of zeros in D, which is the “proportion of agreement”
3. End of loop

The result is plotted against the number of cluster n (Fig 3). After n=6, the proportion of cells in agreement decreases below 80% and remain low.


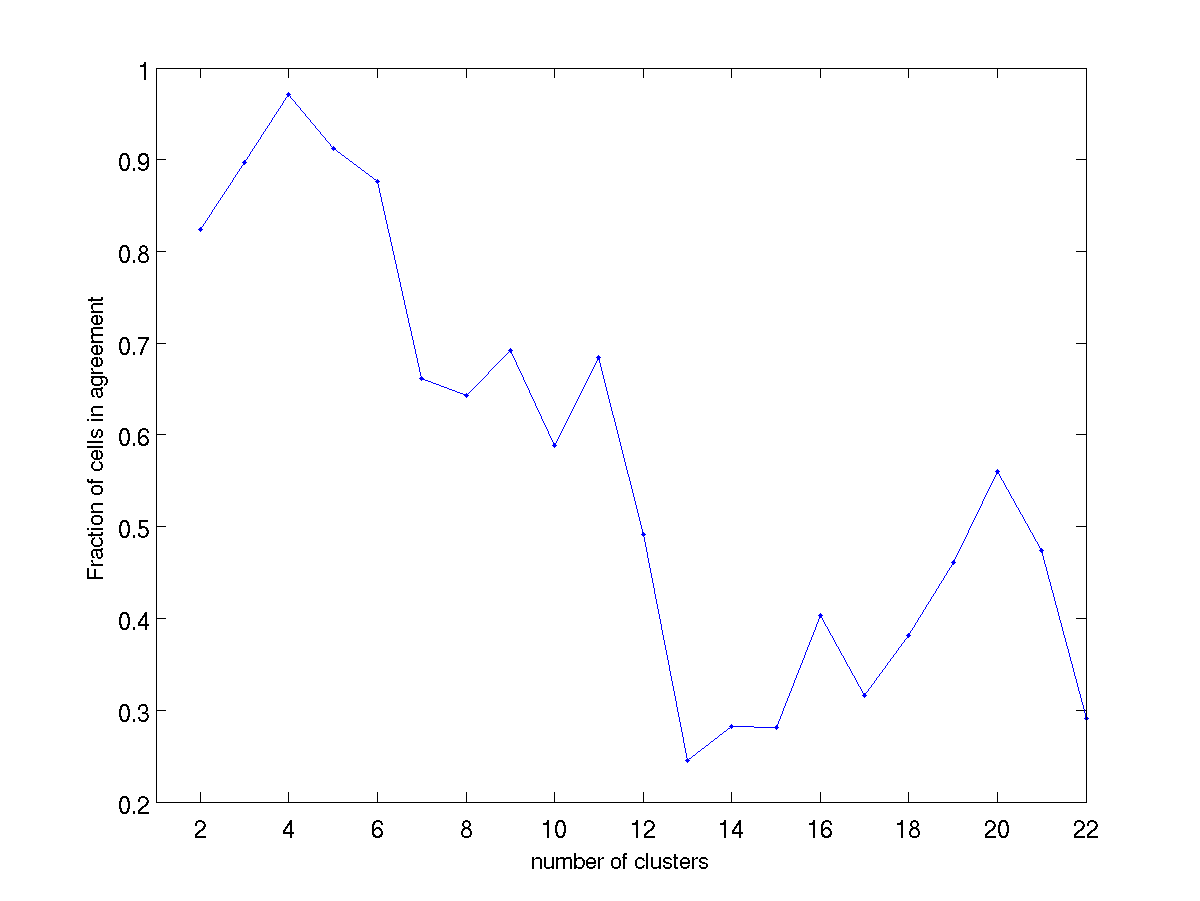


Figure 3 Proportion of agreement vs the number of clusters.
